# Advances in biotechnology and clinical therapy in the field of peripheral nerve regeneration based on magnetism

**DOI:** 10.3389/fneur.2023.1079757

**Published:** 2023-03-10

**Authors:** Zheyuan Fan, Xinggui Wen, Xiangdong Ding, Qianqian Wang, Shoushuai Wang, Wei Yu

**Affiliations:** Department of Hand Surgery, China-Japan Union Hospital of Jilin University, Changchun, China

**Keywords:** magnetic nanoparticles, magnetical nanofibers, peripheral nerve regeneration, magnetic field, repetitive peripheral magnetic stimulation

## Abstract

Peripheral nerve injury (PNI) is one of the most common neurological diseases. Recent studies on nerve cells have provided new ideas for the regeneration of peripheral nerves and treatment of physical trauma or degenerative disease-induced loss of sensory and motor neuron functions. Accumulating evidence suggested that magnetic fields might have a significant impact on the growth of nerve cells. Studies have investigated different magnetic field properties (static or pulsed magnetic field) and intensities, various magnetic nanoparticle-encapsulating cytokines based on superparamagnetism, magnetically functionalized nanofibers, and their relevant mechanisms and clinical applications. This review provides an overview of these aspects as well as their future developmental prospects in related fields.

## 1. Introduction

Peripheral nerve injury (PNI) is a major clinical concern, which is caused by the loss of structure or function of peripheral nerves. In developed countries, ~ 13–23 per 100,000 persons are affected by PNI every year (1). It is a common complication in trauma. The regeneration of the peripheral nerve should be improved to recover its function. The core difficulties in nerve regeneration are the directional prolongation of neurites and the proliferation of Schwann cells (SCs), which provide well-functional axons and myelin sheaths. Currently, the studies on peripheral nerve regeneration involve growth factors, nerve conduits, tissue engineering, and genetic engineering (2–5). Since the 1980's, researchers have been continuously studying the effects of magnetism on nerve cells. The magnetic field can induce the orientation of cellular growth. In 1965, Murayama et al. reported that sickled erythrocytes were oriented perpendicular to the magnetic field (6). This was the first study on this phenomenon. Since then, erythrocytes, collagen, fibroblasts, osteoblasts, human glioblastoma A172 cells, SCs, smooth muscle cells, and PC12 cells have been reported to be related to magnetic fields (6–13). In addition, nerve cells have a better growth potential in the magnetic field [16]. Based on the theoretical effects of magnetic fields on nerve cells, biomaterial interventions have shown promising results in cell cultures and animal studies, providing contact guidance for extending neurites or a sustained release of various drugs and growth factors. Moreover, researchers further investigated the magnetic stimulation of peripheral nerves to obtain a better sense and function. This review summarizes the recent studies on the effects of magnetism on peripheral nerves and proposes future developmental directions in this field.

## 2. PNI and regeneration

PNI is a common clinical issue worldwide, and its prognosis varies with the degree of injury. Unlike the central nerve, the peripheral nerve has a limited regenerative capacity after being injured. After PNI, Wallerian degeneration occurs; the cell bodies of neurons swell, dissolving the chromatin and making Nissl's body disappear, which makes the cell body relatively eosinophilic, shifting the nucleus to the periphery. Moreover, SCs and macrophages phagocytize the myelin and axons, thereby degenerating the myelin sheaths at the distal portion of the nerve injury site. In addition to clearing myelin debris, macrophages and SCs also produce cytokines, which enhance axon growth. After clearing debris, regeneration begins at the proximal portion and continues toward the distal stump. New axonal sprouts generate from the Ranvier nodes (14, 15).

Different cells participate in repairing the injured nerves. SCs are arranged longitudinally to form the bands of Büngner, guiding the direction of regeneration and providing a microenvironment, which promotes regeneration. The tips of the regenerated axons, called growth cones, are composed of flat sheets of cellular matrices with finger-like protrusions (filopodia). Actin polypeptides, which can contract to produce axonal elongation, are in filopodia. The growth cones release proteases, which dissolve the matrix on its path, thereby clearing the way for regeneration. The recovery of nerve function depends on the extension of regenerated axons from the injured site to the target organ at the distant portion (14).

## 3. Theoretical study based on magnetism and peripheral nerve regeneration

### 3.1. Properties of magnetic fields and superparamagnetism

Magnetic properties are generated by electrons, spinning around the nuclei of atoms and their axis (16). The magnetic field intensity of a magnetic material is related to its atomic structure and temperature. In some atoms, the magnetic dipoles, arising from the spinning of electrons, do not cancel each other out, thereby creating a permanent dipole. The permanent dipole aligns with the external magnetic field. The magnetism of superparamagnetic materials disappears with the disappearance of the external magnetic field. This provides a basis for strengthening the magnetic field intensity and reducing the adverse effect of a magnetic field (17).

### 3.2. Growth of nerve cells in a magnetic field

Static magnetic fields (SMFs) or pulsed magnetic fields (PMFs) are usually used in the studies of nerve cells. PMFs are safe and efficient to promote nerve regeneration (18–20). SMFs have constant intensity and direction and a frequency of 0 Hz. Recently, studies have been conducted on the effects of SMFs on nerves at the cellular level. Numerous studies showed that the effects of exposure to SMFs on cellular proliferation varied depending on the cell type (21, 22). Moreover, most studies, investigating the effects of SMFs on cell cycle distribution, showed that there was no statistical difference between the exposed and control group (23). There are two necessary pathways for the regeneration of peripheral nerves: the growth of neurites and myelin sheaths. Mann et al. provided new ideas for the correlations between these two enhanced pathways (24). They reported that a slight nuclear magnetic resonance therapy (NMRT) could induce an increase in SCs proliferation; however, its effect on repair-associated genes was not statistically significant. Furthermore, NMRT could not only enhance the neuronal maturation of individual neurons but also encouraged neurite outgrowth. Interestingly, they also showed that NMRT could induce the secretion of neurotrophic and neuritogenic factors in SCs, leading to the survival of dorsal root ganglia (DRG) neurons and neurite outgrowth.

These studies suggested that the differences in growth direction in response to magnetic field direction depended on the cell type. Macias made two coils of individual copper sheets folded into a square coil to establish a PMF (25). DRG showed a growth tendency parallel to the electric field (perpendicular to the magnetic field). Interestingly, Eguchi showed that SCs and collagens exhibited different growth directions under strong SMFs (8-T). The directions of the SCs and collagen groups were parallel and vertical to that of the magnetic field, respectively. Moreover, SCs oriented in the direction vertical to the magnetic field in a mixture of Schwann cells and collagen under 2 h of magnetic field exposure. DRG showed the same growth tendency as that of collagens, thereby providing theoretical support for the use of a magnetic field to guide peripheral nerve regeneration. Other studies demonstrated that fibrin (26, 27), PC12 cells (12), erythrocytes (28), and osteoblasts (9, 29) aligned parallel to the magnetic field. This property of the magnetic field might be significant in selecting different ways to guide peripheral nerve regeneration.

A strong magnetic field has a destructive effect on cells and can cause genetic mutation and DNA damage (30, 31). Liu et al. reported that the high-intensity magnetic field might be unfavorable for the growth of Schwann cells. When PMF was set to a frequency of 50 Hz, different from 5.0 or 10.0 mT (T = tesla), 0.5, 1.0, or 2.0 mT each was safe for the growth of Schwann cells (32). Interestingly, Eguchi reported that SCs and collagen exhibited normal growth under strong SMFs (8-T) (11). The reasons for these dramatic differences have not been explored yet, and it was speculated that the effects of magnetic field intensity on cellular growth were related to the type of magnetic field. SCs could better withstand SMF as compared to PMF.

### 3.3. Magnetic nanoparticles

A major challenge in peripheral nerve regeneration is the local and sustained delivery of bioactive factors. Studies on MNPs showed that the intervention of an external magnetic field was a type of conventional means. Due to good biocompatibility, MNPs can also be used alone as drug-loading tools to promote peripheral nerve regeneration (33). Magnetic liposomes could enhance neurite outgrowth with a nerve growth factor (NGF) through phosphorylated extracellular regulated protein kinases (ERK) 1/2, which simultaneously upregulated the β-tubulin and integrin β1 (34). Hu et al. showed an increased extension of spiral ganglion neurons (SGNs) in MNPs and MNPs + magnetic field groups as compared to those in the control group (2). Mitrea et al. used oral chitosan-functionalized MNPs to promote neural regeneration in the PNI experimental model and obtained satisfactory results (35). The mechanism for this phenomenon might be the release of Fe ions from iron oxide NPs inside the cells, promoting neurite outgrowth. Numerous approaches used MNPs to assist in drug or gene delivery, including liposome or micelle encapsulation, polymer coatings, and direct surface functionalization (16). Other applications of MNPs include the directional migration of cells induced by external magnetic fields (2, 36–39). In a review, Gilbert divided MNP-mediated cellular manipulations into two categories: magnetic cell guidance and magnetic cell transplantation (16). In essence, both these methods are based on the combination of MNPs and target cells induced by external magnetic fields.

To solve the problem of instability, aggregations, and cellular toxicity of MNPs, Qin et al. synthesized a novel nanomedicine composed of NGF-functionalized Au-coated MNPs. They showed that both the static and rotation magnetic groups could significantly increase the number of differentiated cells and neurites, exhibiting longer average neurite length and clearer directionality, as compared to the no magnet group (36). Liu et al. designed a type of fluorescent–magnetic bifunctional superparamagnetic iron oxide nanoparticles (SPIONs) to control the phenotypic stability of repaired SCs (37). The SMF-induced SPIONs group showed a significantly higher mRNA expression level of brain-derived neurotrophic factor (BDNF), glial cell-derived neurotrophic factor (GDNF), oligodendrocyte transcription factor 1 (Olig1), and vascular endothelial growth factor (VEGF) (qRT-PCR and ELISA). Western blot analysis showed that the expression levels of Beclin1 and lc3b in the experimental group were higher than those in the control group, while the expression level of p62 protein was lower, suggesting the activation of autophagy in Schwann cells by the magnetic stimulation SPIONs. Moreover, for their effects on elongation and branching, there was a statistically significant increase in the expression levels of immune-related cytokines and transcription factors associated with repair phenotypes in the SPION + MF groups. *In vitro* experiments also verified the effects of SPIONs on promoting peripheral nerve regeneration. Hu et al. reported that the combination of SGNs with poly-L-lysine-coated SPIONs could promote the extension of neurites and growth along the direction of a magnetic field (2). Huang et al. reported that the number of SCs with ChABC/PEI-SPIONs, which migrated into the astrocyte region, was 11.6- and 4.6-fold higher than that of control groups under the driven effect of a directional magnetic field (40). Raffa et al. observed that PC12 cells also exhibited more neurite extension and orientation under the guidance of MNPs (39).

### 3.4. Biomaterials based on magnetic fibers

Due to the advantageous features of nanofibers and MNPs, numerous researchers have combined MNPs with biodegradable nanofibers to produce magnetic nanofiber scaffolds. Nanofibers have the characteristics of a large surface-to-mass ratio, high porosity, and superior mechanical performance (41–43). Based on electrospinning (ES), there are three techniques for obtaining iron oxide-loaded composite fibers: the introduction of pre-synthesized SPIONs in a polymer solution before ES (used in biodegradable polymers mostly), mixing a precursor before a post-ES process to yield the SPIONs within the fibers, and *in situ* synthesis techniques (44, 45). There are two major drawbacks in the classical precipitation of NPs on the surface of biomaterials, including difficulty in controlling the morphology and aggregation of NPs and the incompatibility of SPIONs formation with a wide range of biopolymers (44). Silanization, polymer brush coating, and grafting have been used for ameliorating the dispersibility of NPs in polymeric matrices (44). Sodium citrate, polyacid, or oleic acid has also been applied for the dispersion of iron oxide NPs. Nottelet et al. combined thiolyne photoaddition with free ligand exchange to anchor SPIONs on the surface of nanofibers, which maintained the initial fiber morphology and avoided the unwanted aggregation of MNPs.

A variety of materials have been used in the preparation of magnetic nanofibers, such as chitosan/poly (vinyl alcohol) (PVA), poly (ε-caprolactone) (PCL), hydroxyapatite (HA), magnetic poly (L-lactic acid) (PLLA), poly (D, L-lactic acid) (PDLLA), and poly (glycolide-co-L-lactide) (PGLA) (46–50). Due to the tunability, porosity, hydrophilicity, capacity for the incorporation of biological factors, and the polymeric nature of hydrogels, they are increasingly used in bone or nerve regeneration, cancer therapy, and tissue engineering (51). Gilbert et al. designed a method to inject the small conduits of aligned fibers within a hydrogel to reduce fiber tangling (52). They observed that SPION could increase the elongation of neurites by 30%. In addition, they also concluded that SPION content in the electrospinning fibers could be set between 2 and 6% by weight to balance the magnetization and fiber diameter, alignment, and/or density. Gilbert et al. also synthesized aligned control PLLA and SPION-grafted PLLA electro-spun fibers to induce DRG growth. They set up three different types of magnetic fields to study the induction of nerve regeneration by magnetic nanofibers under different magnetic fields: SMF, alternating magnetic field, and linearly moving magnetic field. They observed that neurites could extend 24 and 30% farther on SPION-grafted fibers as compared to that of untethered SPIONs on PLLA fibers under the static or alternating magnetic field. Moreover, 40 and 27% longer neurites were observed in the alternating and linearly moving magnetic fields as compared to that in the SMF (53).

In addition to inducing nerve regeneration under a paramagnetic action, the magnetic field can also be used to form aligned columnar structures in biological scaffolds, especially systematically tuning the mechanical properties of hydrogel scaffolds (54–56). Schmidt et al. used polyimide, magnetic alginate microparticles, and glycidyl methacrylate hyaluronic acid to invent a magnetically aligned regenerative tissue-engineered electronic nerve interface (MARTEENI) (57). This new technology combined TEENI technology with magnetically templated scaffolds to overcome the challenges of prosthetic-limb neural-interfacing technology, such as stiffness mismatch, low axonal population sampling, and long-term signal decay. After 6–8 weeks of exposure, MARTEENI groups showed similar results in the expression levels of extracellular matrix (ECM) proteins and axonal density as compared to the autograft groups. Under the induction of an external magnetic field, magnetic nanofibers can accelerate drug release. Ming-Wei Chang et al. reported that magnetic hollow fibers could induce greater drug release as compared to the non-treated samples under AMF (~40 kHz) (58). This might be due to the magnetocaloric effect caused by superparamagnetism. This kind of nanofiber might have a good prospect in drug delivery with precise positioning.

### 3.5. Mechanism of magnetic effects on nerve cells

**Figure 1:**
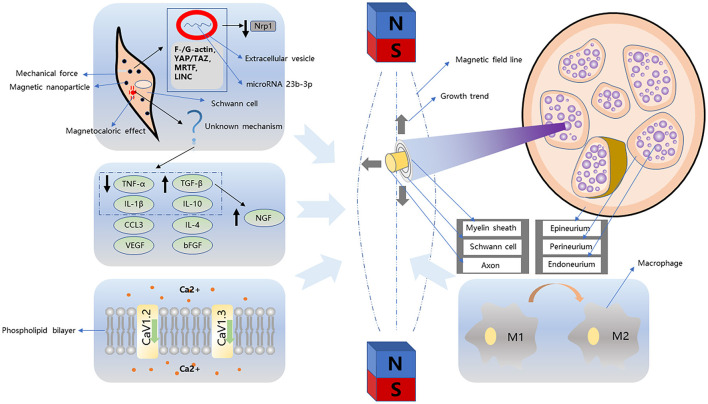
Magnetism-related nerve regeneration strategies can be divided into simple magnetic fields and magnetic nanoparticles. The growth trend of neurites and Schwann cells in the simple magnetic fields is different. Schwann cells tend to grow parallel to the magnetic field, while neurites tend to be vertical. In addition, this process is accompanied by macrophage transformation, cytokine regulation, and changes in ion channels. Strategies based on magnetic nanoparticles generally combine particles with Schwann cells. In this process, Schwann cells are subject to mechanical action and magnetocaloric effect. In the process of mechanical stimulation, Schwann cells are directionally proliferated in the magnetic field by F-/G-actin, YAP/TAZ, MRTF, LINC pathway, and the downregulation of NRP1 induced by microRNA 23b-3p proliferation.

Since the 1970's, increasing evidence has confirmed the neuron-promoting effects of SMF and alternating magnetic fields. The mechanism of the magnetic field, including both external or micromagnetic fields based on superparamagnetism, which affects peripheral nerves, is unclear. A comprehensive understanding of the mechanism at the cellular level might help in developing strategies to use magnet-mediated therapy for PNI. This part of the review highlights the mechanism of stimulating the effects of a magnetic field on cells (Figure 1).

One of the mechanisms of the magnetic field on cells is its effect on the molecular structure of excitable membranes, modifying the function of embedded ion-specific channels (59). Strong magnetic fields can alter the preferred orientation of different diamagnetic anisotropic organic molecules. Low magnetic field intensity relies on the molecular structure of excitable membranes. Researchers have focused on the changes in ion channels under a magnetic field. The voltage-gated channels, including potassium, sodium, and calcium ion channels, are affected by magnetic field exposure, thereby making neurons highly sensitive to magnetic field exposure (60–62). Numerous studies have investigated the internal flow of calcium ions (3, 63). In previous studies, Aldinucci simultaneously exposed human lymphocytes to 4.75 T SMFs and 0.7 mT PMF at 500 MHz for 1 h. The combined SMF and PMF groups could increase the Ca^2+^ influx (64). Balassa et al. reported that the calcium ion channels might play significant roles in synaptic functions under magnetic field stimulation (65). Finberg et al. reported that SMFs could mediate the Ca^2+^ influx through L-type voltage-gated calcium channels (VGCCs), providing a neuroprotective activity related to their anti-apoptotic capacity. They reported that the cellular apoptosis of primary cortical neurons, which were exposed to SMF (50 G), decreased, showing a lower expression level of pro-apoptotic markers, including cleaved caspase-3, cleaved poly ADP ribose polymerase-1, the phospho-histone H2A variant (Ser139), and active caspase-9. Moreover, the expression levels of Cav1.2 and Cav1.3 channels were significantly enhanced (63). Currently, these protective effects on these cells are only reflected in the SMF, while the strong PMF accelerates cellular apoptosis. Prasad et al. reported that the moderate-intensity SMF (0.3 T) could induce the oligodendrocytes precursor cells to increase the intracellular Ca^2+^ influx by increasing the expression levels of L-type channel subunits—CaV1.2 and CaV1.3 (3). Numerous studies showed that the changes in ion channels were an important part of this process.

Studies on MNPs in various magnetic fields have shown that they have great potential for nerve regeneration. After the binding of MNPs to target cells, the magnetic force acting on the target cells becomes the most intuitive mechanism. Schwann cells, which are the commonly used target cells, are essential for the rapid saltatory propagation of action potential. Studies have focused on the effects of mechanical stimulation on Schwann cells (66). Recent studies showed that Schwann cells could create “stimuli” for themselves even in the absence of external mechanical stimulation (67, 68). They secrete basal lamina, an essential component of the SC's ECM, to enhance mechanical resistance. Mechanical stimulation might have a positive impact on SCs based on mechano-sensors and mechano-transducers (66). The stimulation can activate ECM, cell adhesion molecules, and some mechano-transduction pathways (69) and can enhance the expression levels of β1 integrins (70, 71). Moreover, low-level mechanical stimulation can promote the proliferation of SCs accompanied by demyelination and apoptosis (72–74). Xia et al. reported that miR-23b-3p from the extracellular vesicle of SCs might be a key factor of mechanical stimulation, promoting peripheral nerve regeneration (75). Meanwhile, force generation is downstream of many signaling cascades activated by neurotrophic factors, such as netrin-1 and NGF (76, 77). Raffa et al. quantified the correlations between the magnitude of mechanical force and nerve elongation (78, 79). This provided a theoretical basis for the quantitative design of MNPs to promote axonal regeneration.

Another possible mechanism is the magnetocaloric effects of MNPs in an external magnetic field, promoting nerve regeneration. This effect has been applied in bioengineering for a long time. Lin et al. completed and applied the localized heating of magnetic nanofibers to inactivate tumor cells (80). Although there is a lack of studies on the correlation of the magnetocaloric effect with nerve regeneration under a magnetic field, the correlation between temperature and neurite growth has been confirmed. He et al. reported that suitable temperature increased the serum levels of TGF-β and IL-10 and decreased the serum levels of TNF-α and IL-1β (81). This result is the same as the cytokine changes of nerve cells under the magnetic field. Numerous studies proved that the increase in temperature could positively affect the growth of neurites, and the temperature of 37–42°C might be the most suitable temperature for nerve growth (82).

The effects of the magnetic field on peripheral nerves are also reflected in growth factors and inflammatory factors. Zhang et al. reported that the low-intensity PMF could enhance the expression levels of myelin basic protein (MBP), myelin oligodendrocyte glycoprotein expressions (MOG), and transforming growth factor (TGF) (TGF-β1, TGF-βR1, and TGF-βR2) in the central nervous system (83). The TGF-β inhibitor could reduce the expression levels of NGF in a mouse model, which suggested that the stimulation of a magnetic field might promote the release of NGF (4). Mert et al. reported that the low-frequency PMF (1, 3, 5, 7Hz) not only increased compound action potential (CAP) amplitude and sciatic nerve conduction velocity (SNCV) but also reduced the expression levels of chemokines, such as CXCL1 and CCL3, which prevented the infiltration of immune cells and migration of neural progenitors (84). Moreover, PMF treatment could increase the expression levels of bFGF and decrease that of VEGF in the sciatic nerve. Interestingly, VEGF and bFGF indicated different results in diabetic mouse models, showing decreased expression levels (85). The mRNA expression levels of BDNF, GDNF, and VEGF were higher in the magnetic field-induced Schwann cells than those in the control group, while those of *NT-3* were similar (86). There was no reasonable explanation for this phenomenon. It was speculated that this might be related to the differences in the growth directions of Schwann cells and neurites in the magnetic field.

During peripheral nerve regeneration, macrophages gradually convert from the pro-inflammatory (M1) phenotype to the anti-inflammatory (M2) phenotype (87–90). They are also associated with cellular proliferation and differentiation as well as the release of growth factors (91). Dai et al. reported that the combination of MNPs and alternating magnetic fields could promote macrophage to M2 polarization (92). In addition, they also suggested that this regulation might be directly related to the internalization of MNPs involved in activating the expression of interleukin 10 (IL-10), which might be amplified by the external magnetic field.

Brief exposure to SMF at 100 mT for 15 min led to a marked but transient potentiation of binding of a radiolabeled probe for activator protein-1 (AP1) in immature cultured rat hippocampal neurons. It caused a high expression level of growth-associated protein-43 and increased AP1 DNA binding through the expression of Fra-2, c-jun, and jun-D proteins. A study by Hirai provided a basis for finding the signaling pathway of the magnetic stimulatory effects on neurons (93). Liu et al. studied the mechanism of remyelination using *in vitro* and *in vivo* experiments on MNPs bound to SCs under SMFs. They suggested that Raf-MEK-ERK1/2, Rac1-MKK7-JNK-c-Jun, and TORC1-c-Jun pathways might be related to peripheral nerve regeneration under superparamagnetism. Notably, although there was a statistically significant increase for regeneration-related protein in the SPION + MF group as compared to the pure magnetic field group, it was not possible to determine whether this effect was due to superparamagnetism or mechanical stimulation (37).

Numerous high-quality studies are still required to establish a complete system for the mechanistic effects of magnetism on peripheral nerves, which should include all the aforementioned experimental phenomena.

## 4. Clinical applications

With the continuous theoretical studies on peripheral nerve regeneration using a magnetic field, relevant clinical applications are also being developed. Due to its muscle contractions and sensory afferents, repetitive peripheral magnetic stimulation (rPMS) is a non-invasive treatment for the nervous and musculoskeletal system (94) and has been widely used in many fields, such as reducing spasticity and improving the motor control of paretic limbs (95–97).

There is no conclusion about the underlying mechanism of rPMS. Researchers showed that the cortical plastic effects might be a key factor (98). The activation of the frontoparietal loops and an increase in corticomotor excitability are also important effects after magnetic stimulation (96). Liu et al. reported that the peripheral nerve electrical stimulation for 1 h could increase corticomotor excitability and hand dexterity improvement in patients with motor impairment after stroke (99). Other studies have shown similar results (100, 101).

A randomized controlled trial of 46 samples showed that the repetitive magnetic stimulation of the median nerve with 2,400 pulses (20 Hz over 10 min) could increase the peak motor evoked potential (MEP) amplitudes and RC slope in the contralateral hemisphere (98). Furthermore, the improvement in the Purdue Pegboard Test (PPT) score was significant after 24 h as compared to the baseline (^*^P = 0.003) and immediately after rPMS (^*^P = 0.012). Savulescu et al. reported that rPMS in association with physiokinesiotherapy (PKT) groups showed more improved results in the pain score and electromyography (EMG) analysis than in monotherapy groups (102).

rPMS has therapeutic effects on stroke-induced neurological and muscular sequelae. Jiang et al. reported that rPMS groups showed a better arm function and muscle strength for grip and elbow flexion and an extension of patients suffering from an early subacute stroke with severe upper extremity impairment than conventional physiotherapy groups (103). Zschorlich et al. reported that a 5-Hz rPMS could reduce the tendon reflex amplitude, which reduced muscle stiffness and increased mobility (104). Although rPMS involves the direct effect of magnetic field and peripheral nerve, there are few clinical studies on rPMS and peripheral nerve injury. The aforementioned research suggests that rPMS may play a role in promoting peripheral nerve regeneration. We need more high-quality studies to verify the efficacy and specific parameters of rPMS.

Magnetic fields are also applied in chemotherapy-induced peripheral neuropathy (CIPN), which is performed by axonal degeneration (vinca alkaloids) and demyelination (platinum compounds) (105, 106). Rick et al. reported that the 3-month magnetic field treatment could significantly improve NCV and subjectively perceived neurotoxicity (107). Moreover, there was no statistically significant difference in the pain detection end scores.

The International Commission on Non-Ionizing Radiation Protection (lower than 300 Hz for the general public and 400 Hz for occupational exposure) and the Institute of Electrical and Electronics Engineers International Committee on Electromagnetic Safety (lower than 750 Hz) have set limits on electromagnetic field thresholds for protection against stimulation and thermal effects (108–110). Moreover, the threshold of electromagnetic field strength is conservative due to a lack of correlation between internal and external field strengths and nerve activation (108). Hirata et al. used a multi-scale computation to study the threshold of peripheral magnetic stimulation based on a human anatomical model (108). They showed a margin factor of 4–6 and 10–24 times between internal and external protection limits of the international standards.

It is worth noting that the preliminary or explorative studies on the nature of rPMS have reported single cases, case series, or different stimulation protocols (111–115). Sollmann et al. made a checklist, including eight subject-related items, such as age, gender, handedness, or footedness; 16 methodological items, such as coil location, coil type, pulse duration, and shape; and 11 stimulation protocol items, such as stimulation frequency, stimulation intensity, and duty cycle, to guide the research direction of rPMS and contribute to new interventional or exploratory rPMS studies (116).

## 5. Conclusion and future prospects

This review discusses magnetic field properties and intensities, MNP-encapsulating various cytokines based on superparamagnetism, magnetically functionalized nanofibers, relevant mechanisms, and some clinical applications. This review attempted to completely and systematically analyze the latest studies on these topics.

At the same time, there are numerous problems in this field, which should be solved by researchers in their future studies. First, a suitable magnetic field mode and methods to be used are needed to be formulated. Gleich et al. reported that the cornered FO8 coil was more effective, which could reduce the stimulation voltage, current, and energy of the stimulation device (117). Abe et al. also used a newly developed stimulator to reduce the pain and discomfort of magnetic stimulation (118). Heiland et al. reported that the magnetization transfer ratio in the peripheral nerve tissue altered with age (119). This indicated that the mode setting of peripheral nerve magnetic stimulation therapy should be adjusted based on the age of patients.

In addition, the relevant mechanism requires further exploration. The molecular mechanisms of these problems are still unknown. Using experiments based on MNPs-Schwann cells under SMFs, Liu et al. showed that Raf-MEK-ERK1/2, Rac1-MKK7-JNK-c-Jun, and TORC1-c-Jun pathways might be related to peripheral nerve regeneration; however, there is no evidence determining whether this effect was due to superparamagnetism or mechanical stimulation. Moreover, the direction of cell growth was correlated with the cell type under different magnetic fields. According to previous studies, Schwann cells tended to grow parallel to the direction of the magnetic field, while neurites grew perpendicular to the magnetic field. This difference might limit the potential of axon and myelin sheath growth under a magnetic field. Future studies should focus on finding a more reasonable magnetic field placement mode, strength, and time to obtain the best effects with the smallest damage.

The effects of magnetism on peripheral nerves have always been a research hotspot. A suitable application of magnetic field or magnetic biomaterials can shorten the regeneration time of peripheral nerves, enhance the orientation of nerves, and promote the release of growth factors. In future, more high-quality studies are warranted, which could further clarify the mechanism of magnetic action and formulate guidelines for magnetic therapy.

## Author contributions

All authors were involved in the conception and choice of approach for the expert consensus and also contributed findings from their knowledge and experience to the expert consensus, drafted the manuscript, critically revised the manuscript for important intellectual content, and approved the final version of the manuscript.
